# Renal expression and serum levels of high mobility group box 1 protein in lupus nephritis

**DOI:** 10.1186/ar3747

**Published:** 2012-02-20

**Authors:** Agneta Zickert, Karin Palmblad, Birgitta Sundelin, Sangeeta Chavan, Kevin J Tracey, Annette Bruchfeld, Iva Gunnarsson

**Affiliations:** 1 Department of Medicine, Unit of Rheumatology, D2:00 Solna, Karolinska University Hospital, Karolinska Institute, S-171 76 Stockholm, Sweden; 2 Women's and Children's Health, Astrid Lindgren Children's Hospital, Solna, Karolinska University Hospital, Karolinska Institute, S-171 76 Stockholm, Sweden; 3 Department of Pathology and Cytology, Solna, Karolinska University Hospital, Karolinska Institute, S-171 76 Stockholm, Sweden; 4 Laboratory of Biomedical Sciences, Feinstein Institute for Medical Research, 350 Community Drive, Manhasset, NY 11030, USA; 5 Department of Renal Medicine, K56 Huddinge, Karolinska University Hospital, Karolinska Institute, S-141 86 Stockholm, Sweden

## Abstract

**Introduction:**

High mobility group box 1 protein (HMGB1) is a nuclear DNA binding protein acting as a pro-inflammatory mediator following extracellular release. HMGB1 has been increasingly recognized as a pathogenic mediator in several inflammatory diseases. Elevated serum levels of HMGB1 have been detected in autoimmune diseases including Systemic lupus erythematosus (SLE). However, the local expression of HMGB1 in active lupus nephritis (LN) is not known. Here we aimed to study both tissue expression and serum levels of HMGB1 in LN patients with active disease and after induction therapy.

**Methods:**

Thirty-five patients with active LN were included. Renal biopsies were performed at baseline and after standard induction therapy; corticosteroids combined with immunosuppressive drugs. The biopsies were evaluated according to the World Health Organization (WHO) classification and renal disease activity was estimated using the British Isles lupus assessment group (BILAG) index. Serum levels of HMGB1 were analysed by western blot. HMGB1 expression in renal tissue was assessed by immunohistochemistry at baseline and follow-up biopsies in 25 patients.

**Results:**

Baseline biopsies showed WHO class III, IV or V and all patients had high renal disease activity (BILAG A/B). Follow-up biopsies showed WHO I to II (*n *= 14), III (*n *= 6), IV (*n *= 3) or V (*n *= 12), and 15/35 patients were regarded as renal responders (BILAG C/D).

At baseline HMGB1 was significantly elevated in serum compared to healthy controls (P < 0.0001). In all patients, serum levels decreased only slightly; however, in patients with baseline WHO class IV a significant decrease was observed (P = 0.03). Immunostaining revealed a pronounced extranuclear HMGB1 expression predominantly outlining the glomerular endothelium and in the mesangium. There was no clear difference in HMGB1 expression comparing baseline and follow-up biopsies or any apparent association to histopathological classification or clinical outcome.

**Conclusions:**

Renal tissue expression and serum levels of HMGB1 were increased in LN. The lack of decrease of HMGB1 in serum and tissue after immunosuppressive therapy in the current study may reflect persistent inflammatory activity. This study clearly indicates a role for HMGB1 in LN.

## Introduction

Systemic lupus erythematosus (SLE) is a chronic inflammatory autoimmune disease characterized by multiple organ involvement, production of autoantibodies to nuclear components, and immune complex deposition [1]. Lupus nephritis (LN) is regarded as one of the most severe organ manifestations of SLE, affecting approximately 35% to 50% of patients with lupus. Despite increased knowledge of pathogenesis and improved treatment regimens, LN remains a main cause of morbidity among patients with SLE [2].

High-mobility group box 1 protein (HMGB1), a nuclear protein found in all mammalian cells, is known as a DNA-binding protein participating in chromatin structure and transcriptional regulation [3,4]. Extracellular HMGB1 has been identified as a proinflammatory mediator and, owing to its proinflammatory and immunostimulatory properties, has been proposed to contribute to the pathogenesis of multiple chronic inflammatory and autoimmune diseases [5-8]. HMGB1 is actively secreted from activated immune cells such as macrophages and monocytes and is passively released from injured or necrotic cells. When translocated from the nucleus to the extracellular milieu, HMGB1 can act as an 'alarmin', a danger signal that can activate the immune system and has been demonstrated as a key factor in necrosis-induced inflammation [9,10]. Moreover, HMGB1 induces other cytokines such as tumor necrosis factor and interleukin-1 (IL-1), IL-6, and IL-8 and is an activator of endothelial cells leading to the upregulation of adhesion molecules [11,12].

Elevated serum levels of HMGB1 have been found in different inflammatory conditions such as sepsis [13], rheumatoid arthritis [14,15], anti-neutrophilic cytoplasmatic antibody (ANCA)-associated vasculitis [16], and chronic kidney disease [17] as well as in SLE [18-20]. Previous studies have shown increased HMGB1 expression in skin lesions of patients with SLE [21], thus indicating that HMGB1 may be an important mediator of inflammation in target organs in lupus.

Interestingly, increased levels of HMGB1 were recently demonstrated in patients with active LN in comparison with patients with active non-renal disease [22]. However, specified data on the severity of renal disease, histopathological findings, or tissue expression were not included in that study. The aim of this study was to investigate renal tissue expression and serum levels of HMGB1 in correlation not only with renal histopathological and clinical activity but also with response to therapy in order to further investigate its role in patients with LN.

## Materials and methods

### Patients

Thirty-five patients with SLE and biopsy-proven active LN during the period of 1996 to 2008 were included in this study. All patients fulfilled the 1982 American College of Rheumatology classification criteria for SLE [23] and participated in a prospective control program for LN at the rheumatology clinic of Karolinska University Hospital in Stockholm, Sweden. Thirty of the 35 patients were female (86%) and five were men (14%), and the mean age was 34 years (range of 18 to 50). The patients were treated, in accordance with standard therapy for LN, with corticosteroids combined with cyclophosphamide (*n *= 21), mycophenolate mofetil (*n *= 8), or rituximab (*n *= 5), and one patient was treated with azathioprin. After induction therapy for a mean duration of 8 months (range of 6 to 14), all patients underwent a second renal biopsy. At both biopsies, clinical data and blood and urinary samples were collected. Serum samples were frozen at -70°C for future analyses. As a control group for serum levels of HMGB1, blood samples from 48 healthy controls were used [17]. The clinical characteristics of patients and nephritis data at baseline and follow-up are presented in Table 1.

**Table 1:** Clinical characteristics of all patients at baseline and follow-up

	Baseline	Follow-up
Creatinine, μmol/L	92.3 (46.6)	81.6 (42.6)
Proteinuria, g/day	2.0 (1.8)	0.7 (0.8)
C3, g/L	0.53 (0.23)	0.80 (0.23)
C4, g/L	0.09 (0.06)	0.13 (0.07)
C1q	66.6% (41.0%)	75.2% (29.5%)
Anti-dsDNA-positive^a^	83%	44%
Glomerular filtration rate	80.7 (29.4)	88.6 (26.4)
U-erythrocytes > 1+	79%	46%
Renal histology, number		
Class I	15	1
Class II	12	13
Class III	2	6
Class IV	6	3
Class III/V		12
Class V		
Activity index	5.7 (3.1)	2.5 (2.3)
Chronicity index	1.8 (2.0)	2.3 (2.3)
Treatment, number		
Cyclophosphamide	21	
Mycophenolate mofetil	8	
Rituximab	5	
Azathioprin	1	
BILAG renal index, number		
A	32	2
B	3	18
C		7
D		8

Values are presented as mean (standard deviation) unless otherwise indicated. ^a^Anti-double-stranded DNA (anti-dsDNA) raised to at least 1:25. BILAG, British Isles lupus assessment group.

### Evaluation of renal function, histopathology, and renal activity

Renal evaluation at the time point of the first and second biopsies included urine analyses (dipslide procedure), urinary sediment assessment, and investigation of 24-hour urine albumin excretion. Renal function was determined by serum creatinine levels (expressed as micromoles per liter) and estimated glomerular filtration rate (GFR) by using the modification in diet in renal disease (MDRD) formula [24].

Renal biopsies were performed by percutaneous ultrasonography-guided puncture. The renal tissue obtained was evaluated by light microscopy, immunofluorescence, and electron microscopy. The biopsies were graded according to the World Health Organization (WHO) classification of nephritis [25] and additionally scored for activity and chronicity indices [26]. Renal tissue from an unaffected part of a kidney that was nephrectomized owing to carcinoma was used as control. Evaluation of renal activity was estimated by using the British Isles lupus assessment group (BILAG) index [27,28]. Patients with renal BILAG C or D at follow-up were regarded as responders (as previously suggested [29]).

### Serology and complement measures

Assessments of serum IgG anti-double-stranded DNA (anti-dsDNA) antibodies were carried out by immunofluorescence microscopy by using *Crithidiae luciliae *as a source of antigen. Analyses of complement component C1q were performed by rocket electrophoresis by using rabbit anti-C1q as the antibody. Levels of C1q were expressed as the percentage of the levels of healthy blood donors (normal range of 76% to 136%). C3 (normal range of 0.67 to 1.43 g/L) and C4 (normal range of 0.12 to 0.32 g/L) levels were determined by nephelometry.

### HMGB1 serum determination

HMGB1 was analyzed by Western blot in sera from 20 of the patients at baseline and at repeat biopsy by a method previously described [17].

### Immunohistochemical staining of renal biopsies

Immunohistochemical stainings of HMGB1 expression were performed on formaldehyde-fixed paraffin-embedded serial 4-μm sections of renal biopsies. Slides were deparaffinized in xylene and rehydrated with ethanol. Antigen retrieval treatment prior to staining was omitted to allow clearer visualization of extranuclear HMGB1. To block endogenous peroxidase activity, sections were treated with 3% H_2_O_2 _followed by a serum block with 2% human AB sera for 30 minutes and an avidin/biotin-blocking step (Vector Laboratories Inc., Burlingame, CA, USA). The slides were thereafter incubated overnight with an affinity-purified monoclonal mouse IgG2b anti-HMGB1 antibody (concentration of 10 μg/mL, 2G7; Critical Therapeutics Inc., Lexington, MA, USA). A biotin-labeled horse anti-mouse antibody (Vector Laboratories Inc.) containing 2% normal horse serum was used for detection. Stainings were developed by using a DAB kit (Vector Laboratories Inc.) in accordance with the instructions of the manufacturer. Sections were counterstained with Mayer's hematoxylin. Phosphate-buffered saline supplemented with 0.1% saponin was used in all subsequent washes and incubation steps in order to permeabilize the cells. In each assay, controls for specificity of the HMGB1 staining were included on the basis of parallel staining studies omitting the primary antibody, and a primary isotype-matched immunoglobulin of irrelevant antigen specificity (negative mouse IgG2b control; Dako Cytomatation, Glostrup, Denmark) was used. The specificities of extracellular and intracellular HMGB1 immunoreactivities were further verified by blocking experiments with preabsorption of the HMGB1-specific antibody with recombinant HMGB1 prior to staining.

### Statistics

For comparisons of variables at baseline and at repeat biopsy, the non-parametric Wilcoxon matched pair test was used. For comparisons of variables between groups, the non-parametric Mann-Whitney test was used. Correlations were calculated by using Spearman's rank correlation. *P *values of less than 0.05 were considered statistically significant. Statistical evaluation was made with statistical software (STATISTICA 9; StatSoft, Inc., Tulsa, OK, USA).

### Ethics

Informed consent was obtained from all subjects, and the study protocol was approved by the regional ethics committee in Stockholm.

## Results

### Histopathology and renal activity

All patients had an active nephritis at baseline; biopsies showed WHO class III (*n *= 15), III/V (*n *= 2), IV (*n *= 12), or V (*n *= 6). All patients had high renal disease activity; 32 out of 35 had renal BILAG A and three out of 35 a BILAG B. Follow-up biopsies showed WHO I (*n *= 1), II (*n *= 13), III (*n *= 6), IV (*n *= 3), or V (*n *= 12). Two out of 35 patients had renal BILAG A, 18 out of 35 BILAG B, seven out of 35 BILAG C, and eight out of 35 BILAG D. Fifteen patients were thus regarded as responders and 20 out of 35 were regarded as non-responders according to BILAG. The mean renal activity index decreased significantly from 5.7 to 2.5 (*P *< 0.001), whereas no significant findings regarding chronicity index were observed (data presented in Table 1).

### HMGB1 serum determination and evaluation of renal activity

**Figure 1:**
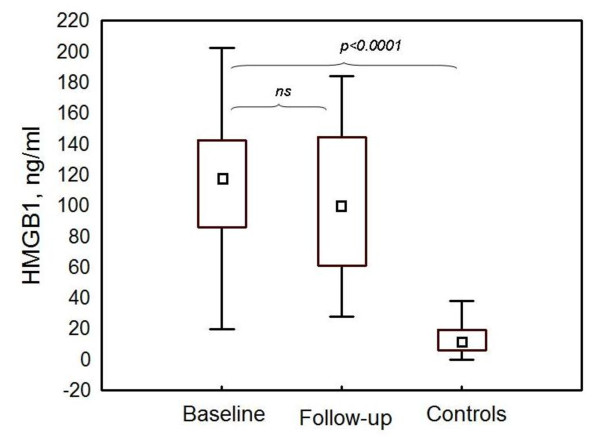
**HMGB1 was significantly elevated in serum of lupus nephritis patients compared with healthy controls**. Serum levels of HMGB1 (expressed as nanograms per milliliter) at baseline and follow-up in lupus nephritis patients (*n *= 20) and controls (*n *= 48). Data are expressed as medians, boxes show 25th to 75th percentiles, and median values are marked inside the boxes. HMGB1, high-mobility group box 1 protein; ns, not significant.

**Figure 2:**
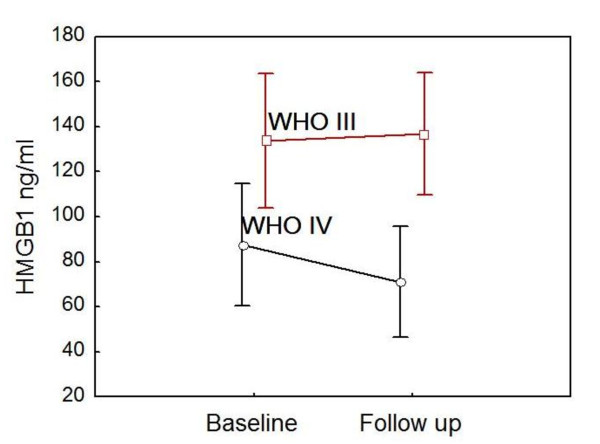
**Serum HMGB1 levels in different World Health Organization (WHO) classes**. Serum levels at baseline and follow-up in lupus nephritis patients with WHO III (*n *= 9) versus IV (*n *= 11) are shown. After induction therapy, serum levels of HMGB1 were significantly decreased in patients with WHO class IV. However, HMGB1 levels remained unchanged in patients with WHO class III. Data are expressed as means, and vertical bars denote 95% confidence intervals. HMGB1, high-mobility group box 1 protein.

At baseline, HMGB1 was significantly elevated in the patient group (mean of 108.4 ± 48.0 ng/mL, median of 117.1, range of 19.8 to 202.4) in comparison with healthy controls (mean of 13 ± 10 ng/mL, median of 12.0, range of 0 to 38.0) (*P *< 0.0001) (Figure 1). Serum HMGB1 levels were significantly higher in the patients with WHO class III (mean of 133.8 ± 47.2 ng/mL, median of 130.0, range of 29.6 to 202.0) in comparison with those with class IV (mean of 87.7 ± 39.1 ng/mL, median of 87.7, range of 19.8 to 155.9) (*P *= 0.01). When all patients were examined, there was at trend toward slightly decreased HMGB1 levels at follow-up (mean of 101.7 ± 50.7 ng/mL, median of 99.7, range of 27.9 to 183.9) (*P *value not significant), although the levels remained significantly high in comparison with those of the control group (*P *< 0.0001) (Figure 1). In the patients with WHO class III at baseline biopsy, no difference in HMGB1 levels was observed at follow-up (mean of 136.9 ± 42.9 ng/mL, median of 142.7, range of 40.3 to 183.9), whereas in class IV, a significant decrease was found (mean of 71.0 ± 35.6 ng/mL, median of 71.4, range of 27.9 to 145.9) (*P *= 0.03) (Figure 2).

**Figure 3:**
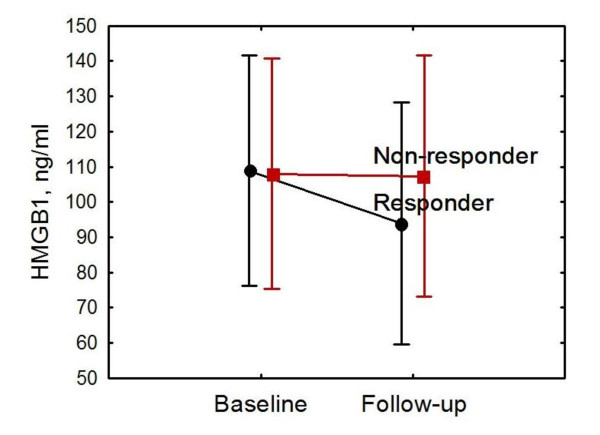
**Serum levels of HMGB1 of responders and non-responders at baseline and follow-up**. Patients with renal BILAG (British Isles lupus assessment group) C or D at follow-up were regarded as responders. In non-responding patients, serum levels of HMGB1 remained high at follow-up. Though not statistically significant, a clear trend of decreasing serum HMGB1 was documented in patients who responded to induction therapy. Data are expressed as means, and vertical bars denote 95% confidence intervals. HMGB1, high-mobility group box 1 protein.

The responder patients (BILAG C/D) had lower serum levels of HMGB1 at follow-up (mean of 93.7 ± 54.2 ng/mL, median of 94.0, range of 27.9 to 181.5) versus those with persisting renal BILAG A or B (mean of 107.4 ± 49.0 ng/mL, median of 108.8, range of 40.3 to 183.9), but the difference was not statistically significant (Figure 3). Though not statistically significant, the serum levels were higher at follow-up in the group of patients (*n *= 4) who developed membranous nephritis (WHO V) in comparison with other WHO classes at follow-up biopsy. There was an inverse correlation between activity index at baseline biopsies and HMGB1 levels both at baseline (*r *= -0.61, *P *< 0.05) and at follow-up (*r *= -0.58, *P *< 0.05). HMGB1 showed no significant correlation with complement levels, creatinine, GFR, proteinuria, or anti-dsDNA antibody positivity (data not shown). Data on serum levels of HMGB1, WHO classification, proteinuria, and renal function are presented in Table 2.

**Table 2:** Serum levels of HMGB1 in 20 patients

	Baseline						Follow-up					
Patient	HMGB1	WHO	AI	CI	U-prot	Crea	HMGB1	WHO	AI	CI	U-prot	Crea
1	19.8	IV	8	0	0.0	66	34.1	I	0	0	0.0	78
2	84.8	IV	8	1	0.2	74	60.6	III	9	1	0.3	78
3	88.4	IV	12	1	1.9	185	89.6	IV	4	3	1.6	86
4	37.1	IV	9	1	3.7	89	27.9	II	2	0	0.9	86
5	29.6	III	5	0	1.3	77	40.3	V	1	2	1.6	87
6	87.9	IV	13	3	1.0	94	71.4	III	4	3	0.4	86
7	86.8	IV	6	0	2.1	64	29.3	II	1	1	3.4	60
8	98.1	IV	12	6	4.9	284	72.6	II	2	8	1.0	306
9	125.9	IV	6	2	1.5	167	109.8	II	1	6	0.1	72
10	120.4	IV	3	0	1.4	90	77.9	II	2	4	0.1	78
11	113.8	III	7	0	1.0	129	128.1	II	1	1	0.1	105
12	126.4	III/V	3	0	3.5	52	129.7	V	2	2	0.7	54
13	130	III	5	1	0.1	70	142.7	II	0	4	0.0	48
14	59.4	IV	6	1	0.3	82	61.7	IV	10	1	2.1	80
15	128.2	III	5	1	1.8	66	148.4	II	1	1	1.8	74
16	155	III	5	5	0.8	53	117.9	II	2	0	0.3	61
17	155.9	IV	5	0	4.8	96	145.9	V	1	0	0	74
18	159.9	III/V	4	0	0.2	53	181.5	V	1	0	0	68
19	158.9	III	5	0	0.3	52	159.9	III	3	1	0.1	66
20	202.4	III	5	1	1.1	60	183.9	III	4	1	0.5	68

Serum levels of high-mobility group box 1 protein (HMGB1) (expressed as nanograms per milliliter), histopathological findings, and renal activity at baseline and follow-up are shown. Creatinine (crea) is expressed as micromoles per liter. AI, activity index; CI, chronicity index, u-prot; 24-hour urine albumin excretion; WHO, World Health Organization.

### Immunohistochemistry

**Figure 4:**
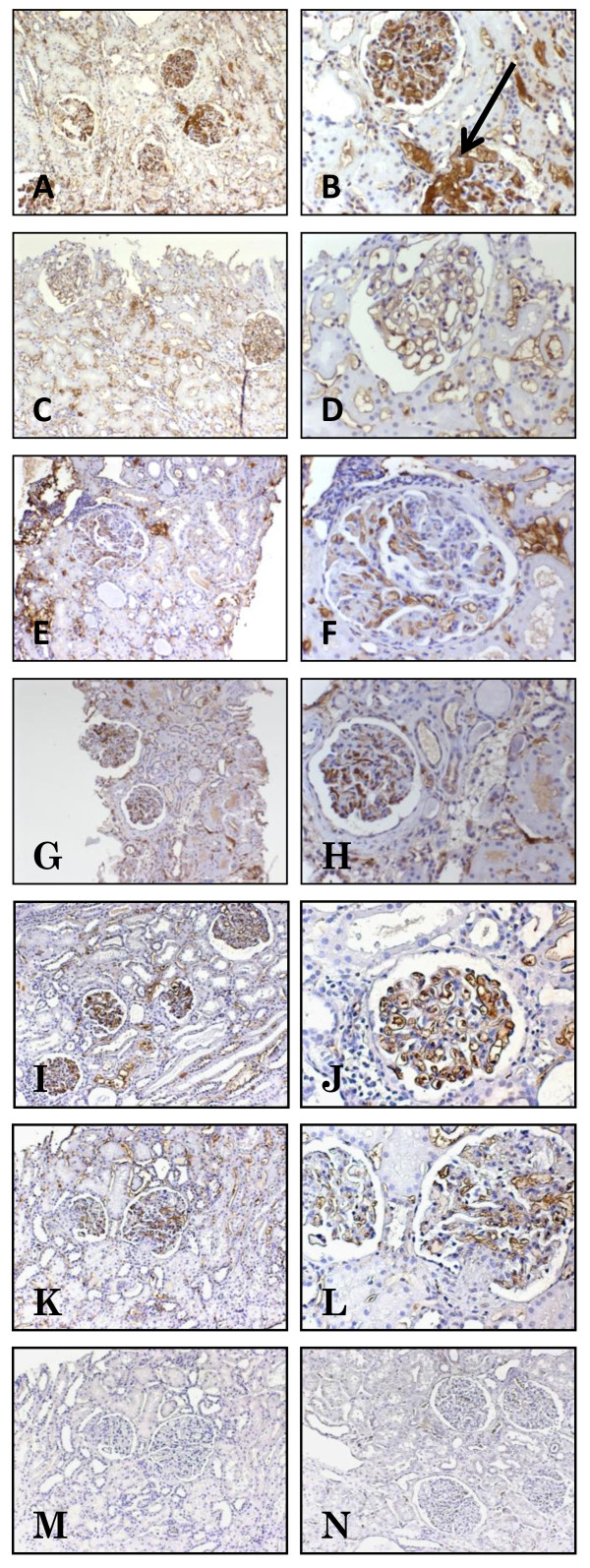
**Expression of extranuclear HMGB1 protein in lupus nephritis (LN)**. Representative micrographs illustrate paraffin-embedded kidney biopsies displaying immunohistochemical diaminobenzidine brown staining of HMGB1. **(A) **A baseline kidney biopsy from a patient with active World Health Organization (WHO) class V LN, magnified in **(B)**, demonstrates that an abundant HMGB1 staining is located predominantly outlining the endothelium but also expressed in the mesangium (arrow). **(C) **The expression of HMGB1 is less pronounced in a repeat biopsy (still classified as WHO V) of the same patient and is magnified in **(D)**. The staining is now most evident outlining the endothelium but is also found in the brush border of tubular cells. **(E) **A baseline biopsy from a patient with active class IV LN, magnified in **(F)**, demonstrates that an abundant HMGB1 expression is located outlining the endothelium. **(G) **A repeat biopsy in a patient with WHO class II after immunosuppressive treatment, magnified in **(H)**, illustrates that HMGB1 is still evident in glomeruli. **(I) **A baseline biopsy from a patient with WHO class III, magnified in **(J)**, demonstrates that an abundant HMGB1 staining is evident predominantly outlining the glomerular endothelium but also expressed in the mesangium. **(K) **HMGB1 expression is still evident in a repeat biopsy (still classified as WHO III) of the same patient, magnified in **(L)**, but is not as strong as in the baseline biopsy. **(M) **A consecutive section from the same patient is stained with irrelevant isotype control antibody. In kidney tissue from a healthy control, HMGB1 expression was predominantly negative or, if present, is restricted to the cell nuclei **(N)**. Original magnifications: 100× (A,C,E,G,I,K,M,N) and 250× (B,D,F,H,J,L). HMGB1, high-mobility group box 1 protein.

Immunostaining revealed an extranuclear HMGB1 expression found in all of the examined biopsies from the patients with LN. The staining was predominantly found outlining the glomerular endothelium and was also documented in the mesangium. A less pronounced HMGB1 expression was documented in vessels and tubular cells (Figure 4). To investigate the amount of HMGB1 expression, an arbitrary scale was used for comparisons between biopsies. Five patients had unchanged amounts, eight had a decrease, and 12 had more abundant amounts of HMGB1 staining. Overall, no difference in the comparison of first and second biopsies was demonstrated (data not shown), and no clear correlation with histopathological classification or clinical outcome could be documented.

HMGB1 is abundantly present in all cell nuclei, thus generating a strong expression that might interfere with visualization of a cytoplasmatic and extranuclear staining, which most often is weaker. By omission of antigen retrieval treatment prior to staining, a clearer visualization of extranuclear HMGB1 expression is allowed. In kidney biopsies from control renal tissue, HMGB1 expression was predominantly negative or, if present, was restricted to the cell nuclei (Figure 4).

## Discussion

To the best of our knowledge, we are the first to demonstrate HMGB1 expression in affected renal tissue from LN patients, in whom we clearly show an increased expression in active disease as well as after immunosuppressive therapy. HMGB1 tissue stainings were predominantly found outlining the glomerular endothelium but also were expressed in the mesangium. In this study, we could also confirm the findings of elevated serum levels of HMGB1 in LN measured by Western blot, as recently shown by Abdulahad and colleagues [22].

We found a clear tissue staining for HMGB1 in LN and this staining was absent in non-lupus control renal tissue. There was no distinct difference in expression of HMGB1 either in the proliferative glomerular lesions or in sites with infiltrates of inflammatory cells in comparison with less affected glomeruli, and the exact origin of the increased renal expression of HMGB1 is not fully clear. The staining outlining the glomerular endothelium could emanate from local extracellular release but also may reflect capture from circulating HMGB1. Thus, one may speculate that the findings of increased serum levels as well as tissue expression of HMGB1 reflect both systemic and local inflammation within the kidney. In glomeruli, the pronounced endothelial staining and the increased expression in the mesangium suggest a co-localization for HMGB1 and immune depositions in LN. However, further studies with other methodologies are required to address this issue.

Elevated HMGB1 expression has previously been demonstrated in lupus patients with cutaneous photoinduced inflammation, in whom cytoplasmatic and extracellular HMGB1 were detected in biopsies from the most clinically active skin lesions in comparison with inactive or non-lesional skin [21]. That study also demonstrated that HMGB1 was still expressed in persisting dermal infiltrates in a proportion of healing lesions, thus suggesting that HMGB1 might be a mediator of both early and late inflammation and may participate in the inflammatory process over longer periods of time.

In a recent study by Bruchfeld and colleagues [16], increased serum levels and renal tissue expression of HMGB1 were demonstrated in ANCA-associated vasculitis with renal involvement. In that study, high baseline serum levels of HMGB1 decreased significantly after treatment, and in contrast to our findings in LN, a more distinct histopathological response and a more distinct clinical response were demonstrated. These findings are consistent with other studies showing that patients with LN, even patients with quiescent clinical pictures, still may have histopathological inflammatory activity after induction treatment [30,31]. Of note, less than half of the patients in the present study were regarded as renal responders according to the definition used. Our observation that serum HMGB1 remained significantly elevated combined with persistent expression in renal tissue at follow-up may reflect an ongoing inflammatory activity in patients with LN despite immunosuppressive therapy. The findings in patients with vasculitis are of particular interest since LN with a focal segmental glomerular pattern (WHO class III), which had the highest HMGB1 levels in our study, has been considered to have features and possibly immune mechanisms similar to those of lesions of systemic vasculitis [32] in comparison with the more widespread global pattern characterized by more pronounced immune depositions seen in patients with class IV. The difference observed in HMGB1 levels when focal and diffuse proliferative nephritis were compared may suggest differences in pathogenic mechanisms and deserves further studies.

In the study by Abdulahad and colleagues [22], a correlation between serum HMGB1 and proteinuria was shown in active SLE patients having some degree of proteinuria but was not confirmed in our study on patients with active biopsy-proven LN only. The two study populations may not be fully comparable, however. Although we could confirm the finding of elevated HMGB1 levels in patients with LN as determined by Western blot, the methods used in the two studies may not be fully comparable (whole serum in the study by Abdulahad and colleagues [22] in comparison with low molecular serum components in the present study). This methodological issue may have contributed to the discrepancies regarding circulating HMGB1 and disease activity between the two studies. Further studies would be of interest to determine the optimal method for HMGB1 determination in patients with LN.

Although HMGB1 levels have been shown to correlate inversely with renal function in patients with chronic kidney disease [17], no such correlation was found in our study or in the previous study of patients with SLE [22]. HMGB1 has been proposed to be involved in the pathogenesis of SLE, and several biological properties of HMGB1 are currently of major interest in SLE research [8]. Inappropriate apoptosis is regarded as a key event in the pathogenesis of SLE. Nuclear autoantigens from not properly cleared apoptotic cells may be excessively presented to the immune system, resulting in loss of self-tolerance and generation of nuclear autoantibodies [33]. HMGB1 released from apoptotic cells may therefore be of special interest in SLE. In primary apoptosis, there is almost no release of HMGB1; however, in conditions with inappropriate clearance of apoptotic material, apoptotic cells may undergo secondary necrosis leading to HMGB1 release [34,35]. HMGB1 has been demonstrated to bind to the chromatin of cells during apoptosis and to remain bound to nucleosomes when released from apoptotic cells. Furthermore, HMGB1-nuclesome complexes have recently been demonstrated to induce the production of proinflammatory cytokines from macrophages and dendritic cells and to induce anti-dsDNA and anti-histone IgG responses and therefore may play a central role in breaking tolerance against nuclear antigens [36]. In that study, circulating HMGB1-nucleosome complexes were detected in serum from patients with SLE but not in healthy controls.

Previous studies have demonstrated high levels of anti-HMGB1 antibodies in patients with SLE [22,37]. This finding raises the possibility that the monoclonal anti-HMGB1 antibody used for tissue staining could have difficulties in binding to HMGB1 if the epitope was already occupied by deposited anti-HMGB1 antibodies. This could possibly contribute to underestimating the local renal expression of HMGB1 and, if so, could explain why there were no clear differences in HMGB1 expression in the most proliferative inflammatory lesions in comparison with less affected glomeruli.

Biomarkers available for assessing renal activity and classification, response to therapy, remission, and flares are insufficient in SLE and do not always reflect the actual inflammatory activity in the renal tissue [30,38]. Although renal biopsy is regarded as the 'gold standard' for assessing renal activity [39], there is a need for new biomarkers for evaluation of disease activity in LN. In the present study, we were not able to determine whether serum levels of HMGB1 could be used for monitoring of renal disease activity *per se *but found persistently elevated levels of serum as well as tissue expression, suggesting that HMGB1 is an important inflammatory mediator in LN. To determine whether a more pronounced reduction of HMBG1 levels can be achieved in a less active phase of LN, more long-term studies with sequential serum HMGB1 determination and repeat biopsies will be required, however.

As therapeutic targeting of HMGB1 has been found to be protective against tissue injury in various preclinical inflammatory disease models (reviewed in [40]), HMGB1 blocking may be of future interest in the development of new treatment strategies in autoimmune disease and also in SLE. As our study was of limited size, additional extended studies will be required to study the role of HMGB1 as a biomarker for renal disease activity in patients with lupus.

## Conclusions

The increased tissue staining for HMGB1, most pronounced outlining the endothelium and in the mesangium, clearly indicates a role for HMGB1 in the inflammatory process of LN. Serum levels of HMGB1 were significantly increased in patients with LN and remained elevated after induction treatment, possibly reflecting persistent inflammatory activity despite immunosuppressive therapy.

## Abbreviations

ANCA: anti-neutrophilic cytoplasmatic antibody; anti-dsDNA: anti-double-stranded DNA; BILAG: British Isles lupus assessment group; GFR: glomerular filtration rate; HMGB1: high-mobility group box 1 protein; IL: interleukin; LN: lupus nephritis; SLE: systemic lupus erythematosus; WHO: World Health Organization.

## Competing interests

The authors declare that they have no competing interests.

## Authors' contributions

AZ participated in patient characterization, acquisition of data, statistical analyses, interpretation of results, and manuscript writing. KP participated in immunohistochemistry staining, interpretation of results, and manuscript writing. BS participated in evaluation of renal biopsies and interpretation of results of HMGB1 tissue staining. SC and KJT were responsible for serum HMGB1 measurement and interpretation of data. AB participated in interpretation of results and manuscript writing. IG participated in study design, interpretation of results, and manuscript writing. All authors were involved in revising the manuscript critically for content and read and approved the final manuscript.
